# Don’t Take It to Heart (Poetry)

**Published:** 1881-11

**Authors:** 


### DON'T TAKE IT TO HEART.

There's many a trouble
Would break like a bubble,
And into the waters of Lethe depart,
Did not we rehearse it,
And tenderly nurse it,
And give it a permanent place in the heart.

There's many a sorrow
Would vanish to-morrow,
Were we not unwilling to furnish the wings
So sadly intruding,
And quietly brooding,
It hatches out all sorts of horrible things.

How welcome the seeming
Of looks that are beeming,
Whether one's wealthy or whether one's poor,
Eyes as bright as a berry,
Cheeks as red as a cheery,
The groan and curse and the heartache can cure.

Resolved to be merry,
All worry to ferry
Across the famed waters that bid us forget;
And no longer tearful,
But happy and cheerful,
We feel life has much that's worth living for yet.

ONCE upon a time in the General Assembly the question was raised of the disposal to be made of the names of church members who could not be found. Some of the commissioners advised that those who did not respond to letters or advertisements should be formally excluded from fellowship. The venerable and eccentric Dr. Samuel Hanson Cox strongly opposed such action as needlessly severe, and said that they might thus excommunicate some who were in heaven.

